# Microscopic cardiac pathology in forensic autopsies: a comparative study of anabolic androgenic steroid users and non-users

**DOI:** 10.1007/s00414-025-03630-y

**Published:** 2025-10-18

**Authors:** Paula Katriina Vauhkonen, Jukka Matti Kiiskilä, Santtu Hytönen, Roosa Koskela, Mikko Ilari Mäyränpää, Katarina Mercedes Lindroos

**Affiliations:** 1 Department of Forensic Medicine, University of Helsinki, P.O. Box 21 (Haartmaninkatu 3) , FI-00014 Helsinki, Finland; 2 Forensic Medicine unit, Finnish Institute for Health and Welfare, P.O. Box 30 (Mannerheimintie 166), FI-00271 Helsinki, Finland; 3 Department of Pathology, University of Helsinki, P.O. Box 21 (Haartmaninkatu 3), FI-00014 Helsinki, Finland; 4 Helsinki University Hospital, Diagnostic center, pathology, P.O. Box 340, FI-00029 Helsinki, Finland

**Keywords:** Forensic autopsy, Anabolic androgenic steroids, Doping, Cardiac pathology, CD3

## Abstract

**Purpose:**

Anabolic androgenic steroid (AAS) abuse is a known risk factor for left ventricular (LV) hypertrophy and cardiac fibrosis. However, these changes are not exclusive to AAS abuse, and similar pathology is often observed in other types of cardiomyopathies. In this study, postmortem cardiac tissue specimens of AAS screened cases were re-examined in detail to determine, whether specific histopathological features could further support the detection of AAS abuse in forensic cause-of-death investigations.

**Methods:**

The sample comprised of 46 Finnish forensic autopsy cases, including 16 AAS positive and 30 AAS negative males, autopsied between 2016 and 2019. Microscopic histopathological features and interstitial lymphocytic inflammation in cardiac tissue were analysed using haematoxylin & eosin (HE), Herovici, and CD3 immunohistochemical staining.

**Results:**

While overall frequencies of arteriolosclerosis, cardiomyocyte hypertrophy, disarray, and fibrosis were similar between the groups, AAS positive cases tended to exhibit more interstitial type LV fibrosis, which was either diffuse or favouring the subepicardial layers. In contrast, right ventricular (RV) fibrosis was absent in AAS positive cases but present in 50% of AAS negative cases. Lymphocytic infiltration was lower in AAS positive cases, with significant differences in LV CD3 + cell densities.

**Conclusion:**

This study highlights that light microscopic examination of cardiac tissue may have limited capacity in distinguishing AAS users from non-users postmortem. However, specific patterns of fibrosis were discovered that may represent histopathological features associated with AAS abuse. Future studies should include larger samples to allow for more robust control of confounding factors and to assess the generalizability of these findings.

**Supplementary Information:**

The online version contains supplementary material available at 10.1007/s00414-025-03630-y.

## Introduction

Sudden deaths of anabolic androgenic steroid (AAS) positive, forensically investigated autopsy cases are most often deemed to be of cardiac origin [1]. These cases typically present with left ventricular (concentric) hypertrophy (LVH), but features of dilated cardiomyopathy (DCM, with CM referring to cardiomyopathy) have also been reported [2]. Microscopically, the most commonly described abnormality is myocardial fibrosis, accompanied by variable degree of cardiomyocyte hypertrophy and disarray, patchy apoptosis or necrosis, and foci of lymphocytic infiltrates [2]. However, based on the scarce available literature, the microscopic features vary from case to case, and similar pathological adaptation is encountered among other autopsy cases with LVH, ischemic and hypertensive CM [3].

Outlining the histopathological features specific for AAS-induced CM would be important not only for the forensic pathologist to help distinguish AAS abuse from other aetiologies of cardiac hypertrophy, but it would also facilitate endomyocardial biopsy interpretation in cases of suspected clinical patient AAS abuse. Specific CM features may also have prognostic importance for clinical patients, as cardiac fibrosis and the degree of inflammatory infiltrations have been found to predict the course of DCM and hypertrophic cardiomyopathy (HCM) [4, 5].

In our previous study, we found that the statistically significant macroscopic cardiac features distinguishing AAS positive forensic autopsy cases from AAS negative control cases were higher heart weight and left ventricular (LV) and septal (S) thickness, even though both samples had macroscopic cardiac hypertrophy [6]. In this study, we re-examined archived cardiac histological specimens of the same study population to determine, if specific microscopic features could further support the detection of AAS abuse in routine forensic casework.

## Materials and methods

### Study sample

The sample included all Finnish forensic autopsy cases that were screened for AAS during the cause-of-death investigation between 2016 and 2019. The sample included 16 AAS assay positive and 30 AAS assay negative Caucasian birth-assigned males (*n* = 46). All the cases underwent a full forensic autopsy, including macroscopic, microscopic, and toxicological investigation, to determine the cause of death at one of the Finnish Institute for Health and Welfare (THL) national Forensic Medicine Units. The initiative for forensic autopsy in Finland is suspected unnatural death (i.e., due to crime, accident, suicide, poisoning), or sudden death without any explanatory disease.

The AAS assays were performed on a request of the forensic pathologists. The results were produced by subcontracted laboratories from postmortem urine samples, via chromatographic mass spectrometric (CG-MS) identification and confirmation of a large scope of available AAS. Demographic characteristics, macroscopic cardiac findings, and causes of death were retrieved from the autopsy reports. A more detailed description of the study sample is available in our previous publications [6, 7].

### Histological samples

The macroscopic autopsy examination included organ weighing, inspection of the coronary arteries, and gross evaluation of complete transverse sections of cardiac tissue. The routine histological sections included 1–5 transmural samples from the left ventricle (LV), 0–2 samples from the septum (S), and 0–2 samples from the right ventricle (RV). All available samples were included in this study. The specimens were fixed in 10% formalin and embedded in paraffin. For this study, one 4 μm section of each sample was cut and stained with haematoxylin & eosin (HE) and a modified Herovici’s stain, according to general procedures. These slides were used for morphological evaluation of cardiac tissue.

Lymphocytic inflammation was evaluated with immunohistochemical staining for labelling CD3 antigen, with EnVision FLEX, High pH kit (Dako), utilizing a polymer-based IHC method. The antibody was visualized with 3,3’-diaminobenzidine (DAB) chromogen and counterstained with haematoxylin followed by permanent mounting. Correspondingly processed autopsy samples of normal tonsil were used as a positive control within each stained batch.

The histological slides were scanned using 3DHISTECH Pannoramic 250 FLASH III digital slide scanner at Genome Biology Unit supported by HiLIFE and the Faculty of Medicine, University of Helsinki, and Biocenter Finland. The images were reviewed by three experienced forensic pathologists, using 3DHISTECH’s CaseViewer 2.4 for Windows [8]. Each slide was evaluated by a single observer. All observers were blinded to the AAS assay result and other data regarding the cause-of-death investigation. This study did not include a formal assessment of intra- or interobserver variability.

The microscopic evaluation included inspection for arteriolosclerosis, cardiomyocyte hypertrophy, cardiomyocyte disarray, fibrosis, and necrosis, according to widely accepted histopathological definitions (histopathological descriptions with representative histology are available in Supplementary Table S1). Due to variability in the number of histological samples per case, each feature was evaluated on a per-case basis. For example, a case was classified as fibrosis-positive if at least one sample demonstrated fibrotic changes, and fibrosis-negative if all the samples were negative for fibrosis. In addition, the anatomical location of each sample (LV, S or RV) was recorded to allow for region-specific subgroup analysis. Fibrosis was further categorized as interstitial, replacement, perivascular, or mixed type; and fibrosis location was defined as subendocardial, myocardial, subepicardial, or a mixture of these (Table S1).

From the CD3 -stained samples, 1–2 transverse sections including epi-, myo- and endocardium (excluding pericardial fat) and a total area of approximately 100 mm^2^ was viewed from each available locus (LV, S, RV). All interstitial CD3 + cell profiles with distinct nucleus were manually annotated, and a total number of positive cell profiles/mm^2^ was calculated for each sample. Cell clusters (hot spots of ≥ 10 CD3 + cells) were separately noted. CD3 + cells within dense fibrotic lesions or vascular lumens were not included in calculations. Six cases were disregarded from CD3 + analysis due to autolytic change, making the samples unreliable for viewing (one AAS assay positive and five AAS assay negative cases; leaving a total sample size of 40 cases for CD3 + calculation).

### Statistical analyses

Statistical analyses were performed with R version 4.3.3 [9]. Differences in frequencies were studied using Fisher’s exact test. Only cases with available data from a given region (LV, S or RV) were included in the region-stratified analyses. The statistical approach for fibrosis type and detailed location was descriptive. Continuous data were assessed with Wilcoxon and Kruskal-Wallis rank sum tests due to the non-normal distributions of the tested variables. Dunn’s test was used for post-hoc calculations for Kruskal-Wallis test, with Bonferroni-corrected p-values *(padj.)*. Statistical significance was set at p-value ≤ 0.05.

## Results

### Study sample demographics and macroscopic cardiac findings

Study sample demographics are summarized in Table 1. Cardiovascular history was unremarkable in the majority of the cases. One AAS positive case had a history of essential hypertension and coronary artery disease (CAD) diagnosed prior to death. In the AAS negative group, one case had been diagnosed with essential hypertension.

**Table 1:** Study sample demographics

		AAS negative (*n* = 30)	AAS positive (*n* = 16)	All (*n* = 46)	*p*-value
Age (years)	Min/Max Med [IQR]	22.5/64.6 33.4 [25.9;45.2]	19.9/53.7 36.6 [30.4;41.8]	19.9/64.6 34.2 [28.5;41.9]	0.387
BMI (kg/m²)	Min/Max Med [IQR]	20.9/59.1 30.1 [27.2;33.0]	23.1/61.6 29.4 [27.2;32.3]	20.9/61.6 29.8 [27.0;32.7]	0.818
Heart weight (g)	Min/Max Med [IQR]	295.0/845.0 445.0 [386.0;487.2]	281.0/865.0 511.5 [438.0;615.8]	281.0/865.0 463.5 [396.0;516.2]	**0.028**
CAD	No	27 (90.0%)	15 (93.75%)	42 (91.3%)	1.000
	Yes	3 (10.0%)	1 (6.25%)	4 (8.7%)	
CV cause of death	No	22 (73.3%)	11 (68.8%)	33 (71.7%)	0.744
	Yes	8 (26.7%)	5 (31.2%)	13 (28.3%)	
Illicit drug/alcohol use	No	11 (36.7%)	5 (31.2%)	16 (34.8%)	0.757
	Yes	19 (63.3%)	11 (68.8%)	30 (65.2%)	

*BMI* Body mass index, *CAD* coronary artery disease, defined by the forensic pathologist, *CV* *cause of death* cardiovascular cause of death, *Illicit drug/alcohol use* stimulants, opioids, cannabinoids and/or alcohol in postmortem toxicology. *P*-values produced with Wilcoxon rank sum test or Fisher’s exact test, when applicable. *Adapted from Vauhkonen et al.2024 *[6],*CC BY 4.0.* *The number and percentage of cases with CV cause of death and illicit drug/alcohol use added*

A detailed description of the macroscopic cardiac findings observed at autopsy, along with the causes of death, is provided in Supplementary Table S2. Significant atherosclerosis in coronary arteries was observed in one AAS positive and three AAS negative cases. In addition, one AAS negative case had left anterior descending artery (LAD) myocardial bridging. Gross evaluation of non-coronary cardiac tissue was unremarkable in 18.8% of the AAS positive and 30.0% of the AAS negative cases. In both groups, concentric and eccentric LV hypertrophy was commonly described. Grossly evident scarring was noted in only one AAS positive and two AAS negative cases; in addition, individual cases were documented to have nonspecific changes, such as scattered pale discoloration, in sectioned tissue. None of the AAS positive cases demonstrated any valve pathologies, while one AAS negative case had aortic valve sclerosis and stenosis (Table S2).

The cause of death was determined to be of cardiovascular origin in 31.2% of AAS positive and 26.7% of AAS negative cases (Table 1, Table S2). Notably, one AAS negative case was diagnosed with unspecified myocarditis. In the majority of the cases in both groups, either alcohol or illicit drugs were discovered in postmortem toxicology (68.8% in the AAS positive and 63.3% in the AAS negative group).

### Microscopic histopathological features of cardiac tissue

The overall frequencies of microscopic cardiac pathology were similar between the groups. Arteriolosclerosis was observed in 31.3% of the AAS positive and 33.3% of the AAS negative cases, cardiomyocyte hypertrophy in 68.8% of the AAS positive and 73.3% of the AAS negative cases, and cardiac fibrosis in 56.3% of the AAS positive and 56.7% of the AAS negative cases. Arteriolosclerosis was mostly notable together with either hypertrophy and/or fibrosis, while hypertrophy occurred with and without fibrosis. Cases with non-fibrotic hypertrophy constituted 25% of the AAS positive and 30% of the AAS negative. Hypertrophied and fibrotic areas generally showed mild, patchy myocyte disarray in both groups. None of the cases had cardiac necrosis. The number of cases declared to have no significant microscopic pathology in any of the available specimens was 3 in the AAS positive group (19%) and 4 in the AAS negative group (13.3%, including two cases with only mild focal arteriolosclerosis). No statistically significant differences emerged in the overall frequencies of these morphological features between the AAS positive and negative cases.

**Fig. 1:**
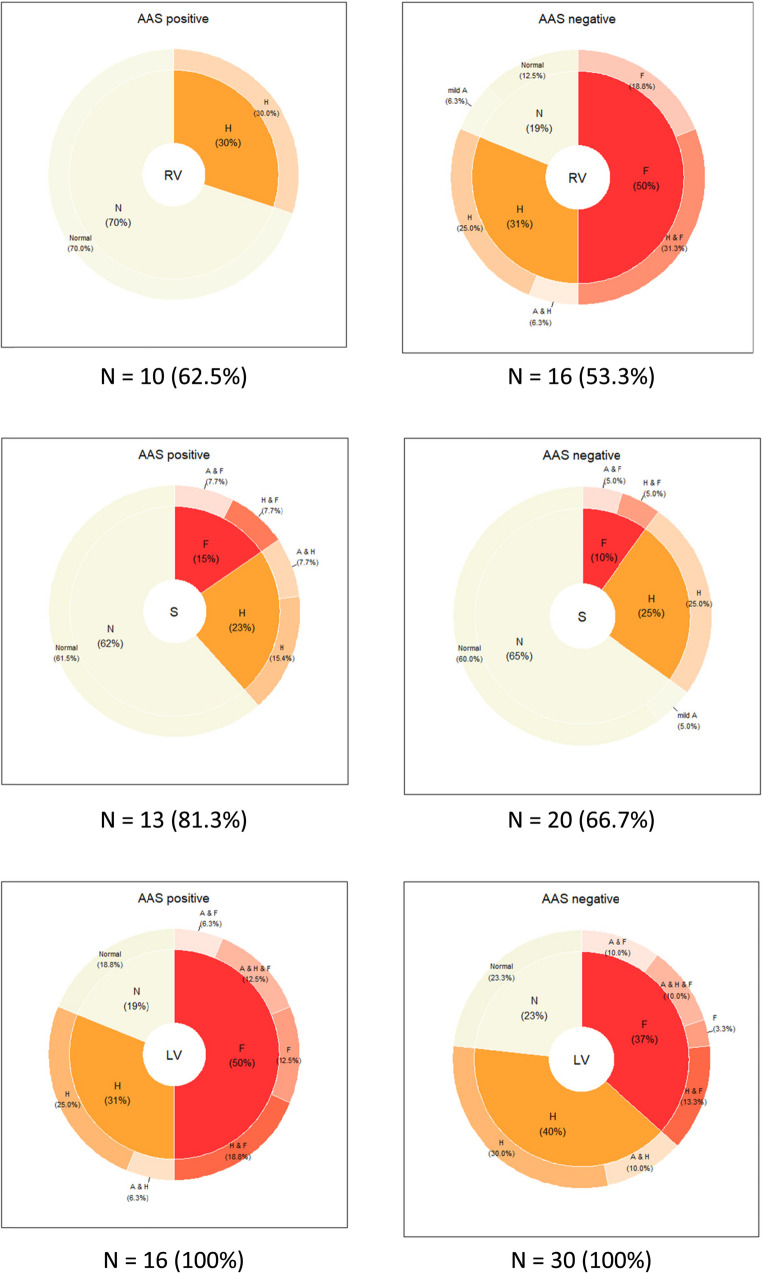
Microscopic cardiac pathology in the AAS positive (n=16) and AAS negative (n=30) groups according to specimen locus. The number (N) and percentage (%) of cases with available specimens are indicated below each pie chart. The inner ring displays aggregated category proportions, rounded to the nearest whole percentage. The outer ring presents subcategory proportions, rounded to one decimal place. RV=right ventricle, S=septum, LV=left ventricle; A=arteriolosclerosis, H=hypertrophy, F=fibrosis, N=no significant pathology

However, when stratified based on the specimen location, only the septal area was similar between the AAS positive and negative groups (Fig. 1). In the AAS positive group, cardiac fibrosis tended to concentrate to the LV (50.0% of cases, vs. 36.7% of cases among the AAS negative; *p* = 0.531), and none of the AAS positive cases had RV fibrosis (compared to 50% of cases in the AAS negative group; *p* = 0.009).

Fibrosis quality and location in each locus is presented in Table 2. Among the AAS positive, LV fibrosis was most often of interstitial type (in two cases this was mixed with a perivascular component), and either diffusely present across all cardiac layers, or favouring the myocardial and subepicardial regions. The opposite was true for the AAS negative, as in these cases fibrosis located more often in subendocardial areas.

**Table 2:** Fibrosis type and location according to specimen locus and AAS status. Values are presented as frequencies (n); percentages (%) are calculated based on the total number of cases with fibrosis in each locus

Locus	Fibrosis type	Fibrosis location	AAS negative	AAS positive
RV	Interstitial	myocardial	4 (50.0%)	0 (0.0%)
	Perivascular	myocardial	1 (12.5%)	0 (0.0%)
	Replacement	-	0 (0.0%)	0 (0.0%)
	Mixed	myocardial	2 (25.0%)	0 (0.0%)
	Mixed	subendocardial	1 (12.5%)	0 (0.0%)
S	Interstitial	myocardial	1 (50.0%)	1 (50.0%)
	Perivascular	subendocardial	1 (50.0%)	0 (0.0%)
	Replacement	-	0 (0.0%)	0 (0.0%)
	Mixed	myocardial	0 (0.0%)	1 (50.0%)
LV	Interstitial	transmural	1 (9.1%)	2 (25.0%)
	Interstitial	subepicardial	0 (0.0%)	1 (12.5%)
	Interstitial	subepicardial & myocardial	0 (0.0%)	1 (12.5%)
	Interstitial	myocardial	2 (∼18.2%)	1 (12.5%)
	Interstitial	myocardial & subendocardial	1 (9.1%)	1 (12.5%)
	Perivascular	subendocardial	3 (∼27.3%)	0 (0.0%)
	Replacement	myocardial & subendocardial	1 (9.1%)	0 (0.0%)
	Replacement	subendocardial	1 (9.1%)	0 (0.0%)
	Mixed	transmural	1 (9.1%)	1 (12.5%)
	Mixed	subepicardial & myocardial	0 (0.0%)	1 (12.5%)
	Mixed	myocardial and subendocardial	1 (9.1%)	0 (0.0%)

*RV* right ventricle, *S* septum, *LV* left ventricle

### Lymphocytic infiltration

**Fig. 2:**
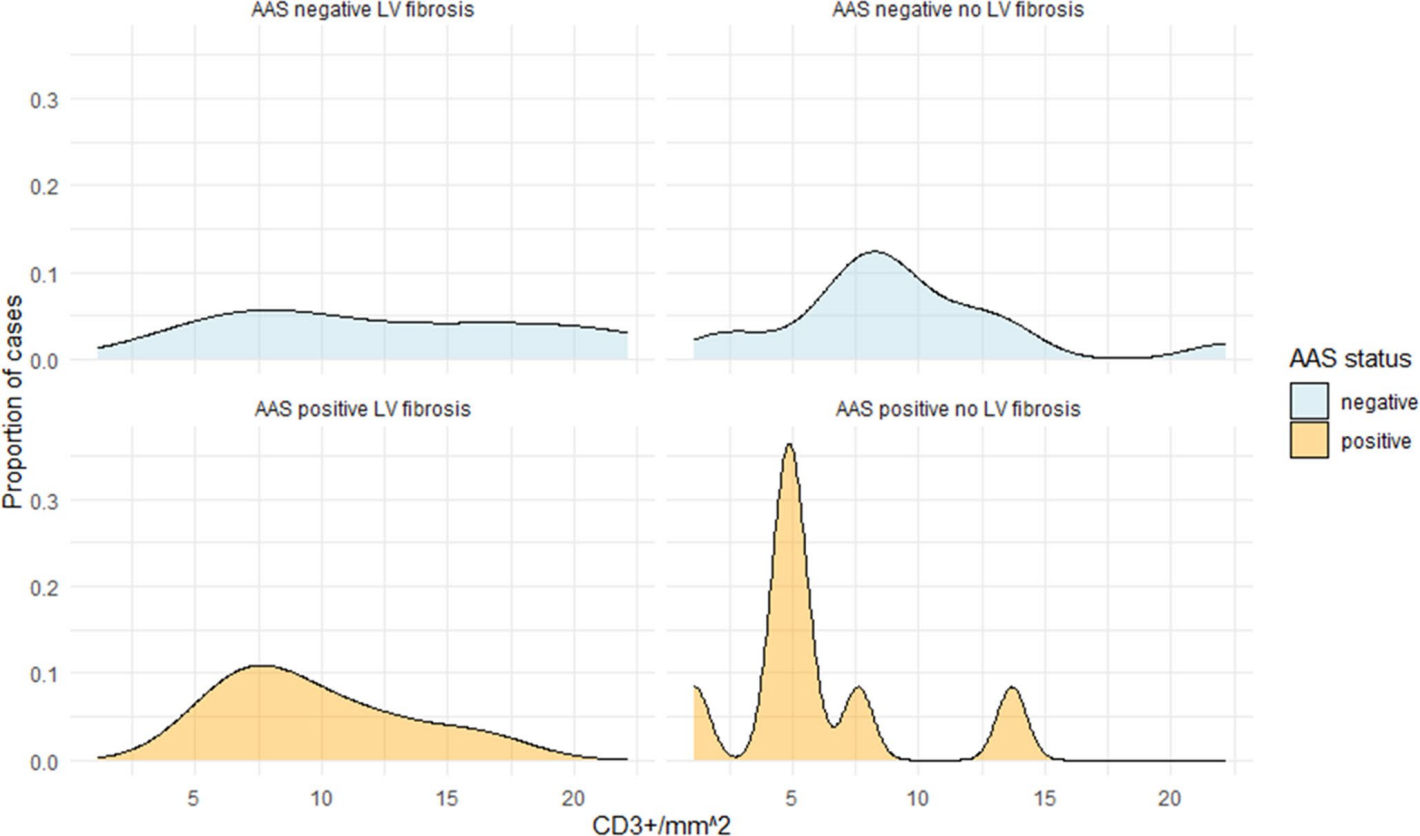
Left ventricle (LV) CD3+ densities according to cardiac fibrosis and AAS status

The degree of CD3 + lymphocytic infiltration in each locus is presented in Table 3. CD3 + densities were systematically lower in the AAS positive group, with a statistically significant rank difference in the left ventricle (*p* = 0.040). The AAS positive also tended to have less lymphocytic clusters. When stratified into groups based on the AAS status (positive/negative) and LV fibrosis (fibrosis/no fibrosis), the difference was found to exist between the AAS negative with LV fibrosis and the AAS positive without LV fibrosis, the latter group having a multimodal density distribution (*padj.=0.013*)(Fig. 2).

**Table 3:** CD3 + densities (positive cells/mm^2^) and lymphocytic clusters (≥ 10 cells) according to specimen locus and AAS status. For clusters, values are presented as frequencies (n); percentages (%) are calculated based on the total number of cases with available specimens in each locus

Locus		CD3 + density (positive cells/mm^2^)		*p*-value^a^	Individual cases with clusters (*n* (%))		*p*-value^b^
		AAS negative	AAS positive		AAS negative	AAS positive	
RV	Min/Max Med [IQR] Mean (std) *N (%)*	3.2/18.7 9.5 [6.3;11.8] 9.4 (4.4) *15 (50.0%)*	1.1/19.9 6.6 [5.2;10.2] 8.1 (5.5) *9 (56.3%)*	0.482	6 (40.0%)	2 (22.2%)	0.657
S	Min/Max Med [IQR] Mean (std) *N (%)*	2.1/27.4 9.5 [6.5;12.1] 10.0 (5.8) *17 (56.7%)*	1.2/12.4 5.9 [4.3;9.1] 6.8 (3.6) *12 (75.0%)*	0.097	7 (41.2%)	3 (25.0%)	0.449
LV	Min/Max Med [IQR] Mean (std) *N (%)*	1.7/22.2 9.0 [6.9;13.7] 10.7 (5.5) *25 (83.3%)*	1.1/16.3 6.2 [4.9;9.3] 7.7 (4.1) *15 (93.8%)*	**0.040**	13 (52.0%)	5 (33.3%)	0.332
All	Min/Max Med [IQR] Mean (std) *N (%)*	3.3/22.2 9.4 [6.9;12.9] 10.3 (5.1) *25 (83.3%)*	1.1/15.0 6.7 [4.7;9.4] 7.6 (4.0) *15 (93.8%)*	0.074	17 (68.0%)	8 (53.3%)	0.502

*RV* right ventricle, *S* septum, *LV* left ventricle, *All* all loci combined

*N(%) *number and percentage of cases with available specimens

^a^Wilcoxon rank sum test, ^b^Fisher’s exact test

## Discussion

In this study, histopathological findings of cardiac tissue in AAS positive autopsy cases were described and compared to AAS negative control cases, with the aim to discover whether light microscopy **–** applied in everyday forensic pathology **–** could be employed as a supplementary tool in identifying antemortem AAS abuse. To our knowledge, this is the first cardio-histopathological study to systematically compare AAS positive autopsy cases to negative ones, produced retrospectively by forensic pathologists blinded to the AAS assay result.

Previous forensic case reports and series suggest cardiac hypertrophy, myocyte disarray, necrosis, patchy lymphocytic infiltrations and fibrosis to be the hallmarks of AAS-induced cardiac injury in light microscopy [2]. Cardiac fibrosis has been reported in a heterogenous manner, with variable descriptions of interstitial, perivascular and replacement -type fibrosis, and some describing these lesions solely as “focal” or “patchy” [1, 2]. The pathophysiology behind these changes is likely mediated via several pathways. First, AAS are capable of inducing cardiomyocyte hypertrophy both directly via the androgen receptor (AR) and indirectly via the Renin-Angiotensin-Aldosterone System (RAAS). The latter pathway may induce LVH and fibrosis due to increased blood pressure, or through angiotensin II and aldosterone -mediated direct effects on the cardiomyocytes [10]. Prolonged AAS abuse has also been associated with increased levels of pro-inflammatory and extracellular matrix remodelling -related markers [11], including interleukin-8, that acts as a direct and undirect chemoattractant to T-lymphocytes [12]. The observed increase in heart mass is thought to arise mainly from AAS-promoted increase in cardiac collagen deposition, rather than mere cardiomyocyte hypertrophy [10, 13]. Part of the AAS-induced cardiac injury is mediated via major ischemic pathways, by acceleration of coronary artery atherosclerosis and increased risk of thrombosis; and via minor ischemic pathways, by promotion of endothelial dysfunction and vasoconstriction. This leads to disturbed coronary microcirculation, and increased susceptibility to reperfusion injury [10, 14–16].

In our study, that predominantly composed of cases with macroscopic cardiac hypertrophy, the microscopic histopathological features of cardiac tissue were quite similar regardless of the AAS assay status. However, there were some differences regarding the distribution and type of cardiac fibrosis. In the AAS positive group, fibrosis was more often located on the LV, as opposed to the S or RV; it was more often of interstitial type; and either transmural or favouring the subepicardial layers. Our observations go nicely with echo cardiac studies indicating an association with AAS abuse and LV cardiac dysfunction [17–20]. These studies have reported both diastolic (reflecting reduced LV compliance) and systolic dysfunction. This dysfunction is generally worse among current AAS users compared to former users; whose cardiac function, in turn, is deteriorated compared to non-AAS using controls. Moreover, the cumulative exposure to AAS seems to be a major determinant of dysfunction severity [20]. These findings together suggest that among the AAS using population, initially subtle cardiac collagen deposition may progress through perivascular and interstitial fibrosis to replacement scars with no regenerative capacity [21]. Some echo cardiac studies have found these pathological changes also extend to the RV in AAS using athletes [22, 23]. In contrast to our results, Nieminen et al. previously reported RV interstitial fibrosis in two endomyocardial biopsy specimens obtained from a sample of three AAS using patients [24]. As the decedents in our sample were on average in their early thirties, their cumulative lifetime exposure to AAS may be shorter than in the previously reported studies. Also, nearly 70% of the AAS positive cases in our sample had died of other causes than CVDs. Our results may thus represent the early stages of AAS-abuse related cardiac damage.

In our study, we found no indication of increased cardiac lymphocytic infiltration in the AAS positive group, and the number of cases with lymphocytic clusters was lower compared to the AAS negative. In general, postmortem lymphocytic counts in cardiac tissue differ from those obtained from clinical patients’ biopsy samples. The CD3 + densities in our study sample fall within previous estimates of “normal” total CD3 + densities in postmortem cardiac specimens without myocarditis [25]. Our results may not be directly extrapolated to the living, but it seems reasonable to hypothesize that cardiac biopsy samples of AAS using patients do not necessarily show signs of CD3 + inflammation. Based on the scarce available literature, frank myocarditis seems infrequent in these cases [26, 27]. Moreover, cardiac necrosis was not observed in the sample. Previously published case reports and series may have included cadavers with more advanced CAD and necrosis due to acute myocardial infarcts, whereas in our sample epicardial CAD was infrequent.

## Limitations

It is important to recognize that this was a national study, in which various forensic pathologists have performed the autopsies. All the available histological specimens were prepared and examined retrospectively. The exact sampling sites for the obtained specimens were not documented, and thus, there may be variation in the specific locations within each locus (LV, S, RV). Also, the small sample size needs to be considered while interpreting the generalizability of our results. In the present study, the ability to detect group differences may be influenced by confounding factors, especially polysubstance use. This presents a significant challenge in forensic research, as polysubstance use commonly co-occurs with AAS abuse. Last, as all the cases in this study were subjected to AAS screening based on the forensic pathologist’s decision and not randomly, prior AAS abuse within the control group cannot be excluded.

## Conclusion

Our study compared the microscopic histopathological features of cardiac tissue in AAS positive and negative forensic autopsy cases. We found that similar pathology occurred in both groups. However, in the AAS positive group, there was a tendency towards LV rather than RV fibrosis. Moreover, LV fibrosis was almost exclusively interstitial, and either diffuse or favouring the subepicardial regions. No indication of increased CD3 + lymphocytic inflammation was observed among the AAS positive in this study. Future studies should aim to include a larger sample size and preferably cases identified through random AAS screening, enabling more robust control for potential confounders and enhancing the generalizability of the findings to a larger forensic autopsy population.

## Supplementary Information

Below is the link to the electronic supplementary material.

Supplementary Material 1 (PDF 496 KB)

Supplementary Material 2 (PDF 220 KB)

## Data Availability

The data underlying this article will be shared on reasonable request to the corresponding author.
