# Supplementary material for: Microscopic cardiac pathology in forensic autopsies: a comparative study of anabolic androgenic steroid users and non-users
Source: Int J Legal Med. 2025 Oct 18;140(2):795–806. doi: 10.1007/s00414-025-03630-y (PMC12956918; doi:10.1007/s00414-025-03630-y)
Supplement: Supplementary file 1 — Supplementary Material 1 (PDF 496 KB) [file 414_2025_3630_MOESM1_ESM.pdf]

Microscopic Cardiac Pathology in Forensic Autopsies: A Comparative Study of Anabolic Androgenic Steroid Users and Non-Users

International Journal of Legal Medicine

Paula Katriina Vauhkonen[a,b], Jukka Matti Kiiskilä[b], Santtu Hytönen[b], Roosa Koskela[b], Mikko Ilari Mäyränpää[c], Katarina Mercedes Lindroos[b]

[a]Department of Forensic Medicine, University of Helsinki, P.O. Box 21 (Haartmaninkatu 3), FI-00014, Helsinki, Finland.

[b]Forensic Medicine unit, Finnish Institute for Health and Welfare, P.O. Box 30 (Mannerheimintie 166), FI-00271, Helsinki, Finland.

[c]Department of Pathology, University of Helsinki, P.O. Box 21 (Haartmaninkatu 3), FI-00014, Helsinki, Finland and Helsinki University Hospital, Diagnostic center, pathology, P.O. Box 340, FI-00029, Helsinki, Finland.

Corresponding author: Paula Katriina Vauhkonen, paula.vauhkonen@helsinki.fi, +358504945329

Supplementary Table S1. Histopathological features evaluated in the study

Representative histopathological images were obtained from the study sample (haematoxylin & eosin stain).

Feature	Histopathological description	Representative histology
**Arteriolosclerosis** [1]	Thickening of small intramyocardial arterioles due to smooth muscle proliferation or hyaline deposits, with subsequent luminal narrowing	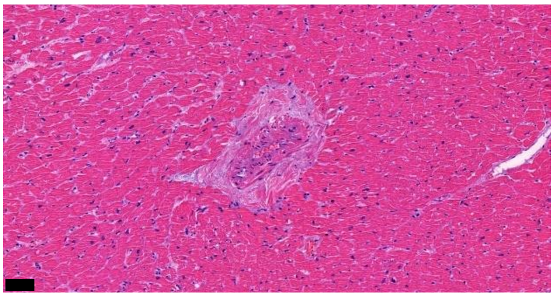 Scale bar = 50 µm
**Cardiomyocyte hypertrophy** [2]	Increase in cardiomyocyte size (> 15 µm in diameter), with nuclear enlargement/hyperchromasia	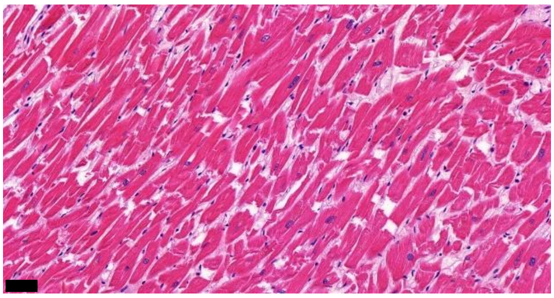 Scale bar = 50 µm
**Cardiomyocyte disarray** [2]	Disorganization of cardiomyocyte architecture	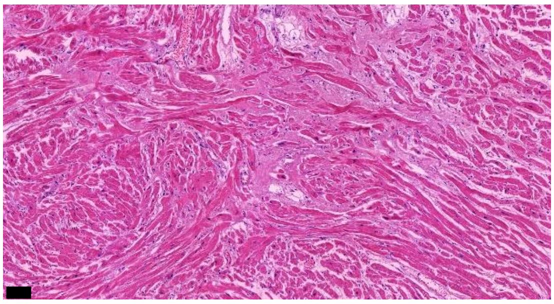 Scale bar = 100 µm

**Myocardial fibrosis** [3]	Abnormal deposition of connective tissue<br><br>**Type**<br><br>• Interstitial, without myocyte loss = interstitial<br>• Interstitial, with myocyte loss = replacement<br>• Perivascular areas = perivascular<br>• More than one type = mixed<br><br>**Location**<br><br>• Adjacent to endocardium = subendocardial<br>• Mid-myocardial = myocardial<br>• Adjacent to epicardium = subepicardial<br>• More than one type = mixed	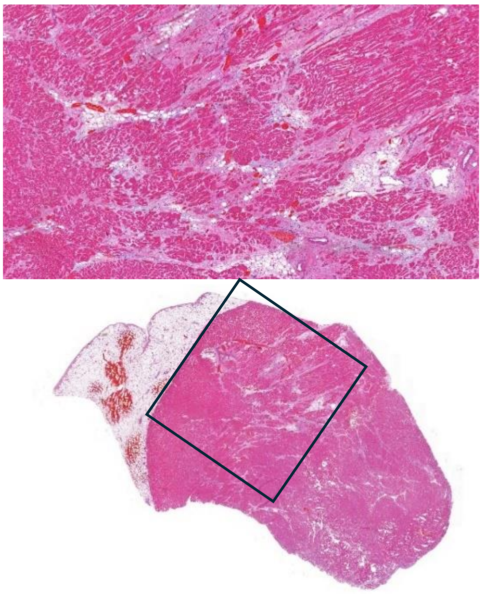<br>*Mixed perivascular and interstitial fibrosis in subepicardial and myocardial regions*
**Myocardial necrosis** [4]	Coagulative necrosis, with or without acute inflammation	Representative image is provided in the cited reference

References (Supplementary Table S1)

[1] Mitchell RN (2018) Heart. In: Kumar V, Abbas AK, Aster JC (eds). Robbins Basic Pathology. 10th edn. Elsevier, Philadelphia, pp 367-368

[2] Basso, C., Michaud, K., d'Amati, G. et al. (2021) Cardiac hypertrophy at autopsy. Virchows Arch 479, 79–94. doi: 10.1007/s00428-021-03038-0

[3] Frangogiannis NG (2021) Cardiac fibrosis. Cardiovasc Res 117(6):1450–1488. doi:10.1093/cvr/cvaa324

[4] Mitchell RN (2018) Heart. In: Kumar V, Abbas AK, Aster JC (eds). Robbins Basic Pathology. 10th edn. Elsevier, Philadelphia, pp 414-416
