# Supplementary material for: Microscopic cardiac pathology in forensic autopsies: a comparative study of anabolic androgenic steroid users and non-users
Source: Int J Legal Med. 2025 Oct 18;140(2):795–806. doi: 10.1007/s00414-025-03630-y (PMC12956918; doi:10.1007/s00414-025-03630-y)
Supplement: Supplementary file 2 — Supplementary Material 2 (PDF 220 KB) [file 414_2025_3630_MOESM2_ESM.pdf]

Microscopic Cardiac Pathology in Forensic Autopsies: A Comparative Study of Anabolic Androgenic Steroid Users and Non-Users

International Journal of Legal Medicine

Paula Katriina Vauhkonen[a,b], Jukka Matti Kiiskilä[b], Santtu Hytönen[b], Roosa Koskela[b], Mikko Ilari Mäyränpää[c], Katarina Mercedes Lindroos[b]

[a]Department of Forensic Medicine, University of Helsinki, P.O. Box 21 (Haartmaninkatu 3), FI-00014, Helsinki, Finland.

[b]Forensic Medicine unit, Finnish Institute for Health and Welfare, P.O. Box 30 (Mannerheimintie 166), FI-00271, Helsinki, Finland.

[c]Department of Pathology, University of Helsinki, P.O. Box 21 (Haartmaninkatu 3), FI-00014, Helsinki, Finland and Helsinki University Hospital, Diagnostic center, pathology, P.O. Box 340, FI-00029, Helsinki, Finland.

Corresponding author: Paula Katriina Vauhkonen, paula.vauhkonen@helsinki.fi, +358504945329

Supplementary Table S2. Description of the macroscopic cardiac findings and the causes of death in the study sample (AAS positive n=16, AAS negative n=30)

Table abbreviations: RCA = Right Coronary Artery, LAD = Left Anterior Descending artery, LCX = Left Circumflex artery, RV=Right ventricle, S=septum, LV=left ventricle, NA = data not recorded in the autopsy report

Case	AAS status	Heart weight (g)	Left ventricle thickness (cm)	Septum thickness (cm)	Right ventricle thickness (cm)	Coronary arteries	Macroscopic description	Underlying cause of death[a]
1	positive	528	1,7	NA	0,4	Fatty streaks and small plaques, no obliteration.	Rounded appearance. LV eccentric hypertrophy. No grossly visible scarring or focal lesions.	Toxic effect of alcohol
2	positive	322	1,8	2,0	0,3	Small plaque in LAD, no obliteration.	Asymmetric LV hypertrophy. No grossly visible scarring or focal lesions.	Poisoning by drugs
3	positive	608	2,0	1,7	0,6	Fatty streaks in LCX, no obliteration.	Pericardial fat increased. Biatrial and biventricular dilatation. No grossly visible scarring or focal lesions.	Poisoning by drugs
4	positive	450	1,9	NA	NA	Fatty streaks and small plaques, no obliteration.	LV hypertrophy. No grossly visible scarring or focal lesions.	Pulmonary embolism
5	positive	500	1,4	2,0	NA	NA	LV hypertrophy. No grossly visible scarring or focal lesions.	Asphyxiation due to hanging

Case	AAS status	Heart weight (g)	Left ventricle thickness (cm)	Septum thickness (cm)	Right ventricle thickness (cm)	Coronary arteries	Macroscopic description	Underlying cause of death[a]
6	positive	485	NA	NA	NA	Fatty streaks, no obliteration.	LV chamber mildly dilated. Focal reddish area in LV anterior wall. RV and LV wall thickness within normal limits.	Poisoning by drugs
7	positive	523	1,9	2,0	0,4	Fatty streaks, no obliteration.	Increased myocardial consistency. No grossly visible scarring or focal lesions.	Poisoning by narcotics
8	positive	722	2,2	NA	0,6	Calcification and severe obliteration of all coronary arteries.	Biatrial and biventricular dilatation. Focal scar in LV anterior wall adjacent to the anterior papillary muscle insertion.	Atherosclerotic heart disease
9	positive	281	1,3	NA	0,4	Fatty streaks in LAD and LCX.	Grossly unremarkable. No visible scarring or focal lesions.	Asphyxiation due to hanging
10	positive	865	2,0	2,0	0,5	Unremarkable.	Global enlargement. Increased myocardial consistency. Patchy hyperaemia in LV free wall.	Cardiomegaly
11	positive	490	NA	NA	NA	Fatty streaks, no obliteration.	Grossly unremarkable. No visible scarring or focal lesions.	Poisoning by drugs
12	positive	550	1,5	1,4	0,3	Unremarkable.	LV eccentric hypertrophy. No grossly visible scarring or focal lesions.	Cardiomegaly

Case	AAS status	Heart weight (g)	Left ventricle thickness (cm)	Septum thickness (cm)	Right ventricle thickness (cm)	Coronary arteries	Macroscopic description	Underlying cause of death[a]
13	positive	645	2,0	2,0	0,3	Unremarkable.	LV concentric hypertrophy, RV chamber dilatation. No grossly visible scarring or focal lesions.	Poisoning by drugs
14	positive	402	3,0	2,0	0,5	Fatty streaks and small plaques, no obliteration.	LV concentric hypertrophy. No grossly visible scarring or focal lesions.	Other specified injuries involving multiple body regions
15	positive	394	1,2	1,2	0,3	Fatty streaks, no obliteration.	Grossly unremarkable. No visible scarring or focal lesions.	Other specified injuries involving multiple body regions
16	positive	639	1,4	1,7	0,4	Unremarkable.	Rounded appearance. No grossly visible scarring or focal lesions.	Cardiomegaly
17	negative	440	NA	NA	NA	Fatty streaks, calcification, mild distal obliteration of all coronary arteries.	Grossly unremarkable. No visible scarring or focal lesions.	Toxic effect of alcohol
18	negative	497	2	1,8	0,4	Fatty streaks in RCA, no obliteration.	Aortic valve sclerosis and moderate stenosis. No grossly visible scarring or focal lesions.	Aortic valve stenosis
19	negative	488	1,7	1,6	0,9	Unremarkable	Global enlargement. No grossly visible scarring or focal lesions.	Pulmonary embolism

Case	AAS status	Heart weight (g)	Left ventricle thickness (cm)	Septum thickness (cm)	Right ventricle thickness (cm)	Coronary arteries	Macroscopic description	Underlying cause of death[a]
20	negative	455	1,6	1,2	0,4	Fatty streaks, no obliteration.	LV hypertrophy, papillary muscle hypertrophy. No grossly visible scarring or focal lesions.	Focal brain injury
21	negative	320	NA	NA	NA	Unremarkable.	Grossly unremarkable. No visible scarring or focal lesions.	Asphyxiation due to hanging
22	negative	410	NA	NA	NA	Unremarkable.	Grossly unremarkable. No visible scarring or focal lesions.	Poisoning by drugs
23	negative	445	1,7	1,5	0,4	Fatty streaks in RCA, calcified plaque in LAD with mild obliteration.	Increased myocardial consistency. No grossly visible scarring or focal lesions.	Hypertensive heart disease with (congestive) heart failure
24	negative	472	1,8	1	0,4	Unremarkable.	LV eccentric hypertrophy. No grossly visible scarring or focal lesions.	Poisoning by drugs
25	negative	485	1,5	1,5	0,5	Unremarkable.	Increased myocardial consistency and pallor in LV.	Mental and behavioral disorders due to multiple drug use
26	negative	295	1	1	0,4	Unremarkable.	Grossly unremarkable. No visible scarring or focal lesions.	Asphyxiation due to hanging
27	negative	471	1,4	1,3	0,4	Unremarkable.	Saggy appearance. No visible scarring or focal lesions.	Poisoning by drugs

Case	AAS status	Heart weight (g)	Left ventricle thickness (cm)	Septum thickness (cm)	Right ventricle thickness (cm)	Coronary arteries	Macroscopic description	Underlying cause of death[a]
28	negative	640	1,8	NA	0,5	Small plaques, no obliteration.	Biatrial and biventricular dilatation. No grossly visible scarring or focal lesions.	Toxic effect of alcohol
29	negative	342	1,5	1	0,5	Unremarkable.	LV concentric hypertrophy. Scattered pale discoloration in LV. Focal hyperaemia in LV posterior wall.	Cardiomyopathy, unspecified
30	negative	384	1,1	1,1	0,4	Unremarkable.	Grossly unremarkable. No visible scarring or focal lesions.	Asphyxiation due to hanging
31	negative	845	1,6	1,4	0,6	Fatty streaks in LCX, no obliteration.	Biatrial and biventricular dilatation. Pallor in LV free wall.	Poisoning by drugs
32	negative	456	1,1	1,1	0,4	Unremarkable.	LV chamber mildly dilated. Scattered discoloration in LV posterior wall.	Pneumonia, unspecified
33	negative	426	1,7	1,2	0,5	Unremarkable.	Rounded appearance. LV concentric hypertrophy. Scattered pale discoloration in RV and LV.	Myocarditis, unspecified
34	negative	302	1,5	1,2	0,5	Unremarkable.	Scattered pale discoloration in LV.	Asphyxiation due to mechanical obstruction of the upper airway
35	negative	413	1,6	1,6	0,6	Unremarkable.	LV eccentric hypertrophy. No grossly visible scarring or focal lesions.	Drowning

Case	AAS status	Heart weight (g)	Left ventricle thickness (cm)	Septum thickness (cm)	Right ventricle thickness (cm)	Coronary arteries	Macroscopic description	Underlying cause of death[a]
36	negative	446	1,9	2,1	0,3	Fatty streaks in RCA, plaque in LAD with significant obliteration.	LV concentric hypertrophy. Macroscopic scar in LV lateral wall.	Old myocardial infarction
37	negative	505	1,3	1,2	0,5	Unremarkable.	LV eccentric hypertrophy. Pallor in LV.	Asphyxiation due to nitrous oxide -induced hypoxia
38	negative	404	1,4	NA	0,4	Unremarkable.	Biventricular dilatation. No grossly visible scarring or focal lesions.	Myocardial fibrosis
39	negative	355	1,4	1,3	0,5	Unremarkable.	LV subendocardial fibrosis.	Toxic effect of alcohol
40	negative	392	1,3	1,2	0,5	Small plaques, no obliteration.	LAD myocardial bridging. No grossly visible scarring or focal lesions.	Drowning
41	negative	530	1,6	NA	0,4	Fatty streaks, no obliteration.	Biatrial and biventricular dilatation. No grossly visible scarring or focal lesions.	Cardiomegaly
42	negative	314	1,2	1,1	0,4	Unremarkable.	Grossly unremarkable. No visible scarring or focal lesions.	Asphyxiation due to hanging
43	negative	343	1,1	1,1	0,4	Fatty streaks, no obliteration.	Grossly unremarkable. No visible scarring or focal lesions.	Toxic effect of alcohol
44	negative	504	2	2	0,3	Unremarkable.	Global enlargement. Pericardial fat increased. No grossly visible scarring or focal lesions.	Poisoning by narcotics

Case	AAS status	Heart weight (g)	Left ventricle thickness (cm)	Septum thickness (cm)	Right ventricle thickness (cm)	Coronary arteries	Macroscopic description	Underlying cause of death[a]
45	negative	520	NA	NA	NA	Fatty streaks, no obliteration.	Grossly unremarkable. No visible scarring or focal lesions.	Other specified injuries involving multiple body regions
46	negative	480	NA	NA	NA	Unremarkable.	Grossly unremarkable. No visible scarring or focal lesions.	Poisoning by narcotics

[a] Determined according to the International Classification of Diseases, 10th Revision (ICD-10) coding system.
